# Components of Mathematics Anxiety: Factor Modeling of the MARS30-Brief

**DOI:** 10.3389/fpsyg.2016.00091

**Published:** 2016-02-17

**Authors:** Belinda Pletzer, Guilherme Wood, Thomas Scherndl, Hubert H. Kerschbaum, Hans-Christoph Nuerk

**Affiliations:** ^1^Department of Psychology, Paris-Lodron University SalzburgSalzburg, Austria; ^2^Department of Psychology, University of GrazGraz, Austria; ^3^Department of Cell Biology, Paris-Lodron-University SalzburgSalzburg, Austria; ^4^Department of Psychology, Eberhard Karls University TübingenTübingen, Germany

**Keywords:** mathematics anxiety, confirmatory factor analysis, Mathematics Anxiety Rating Scale, sex differences, career choice

## Abstract

Mathematics anxiety involves feelings of tension, discomfort, high arousal, and physiological reactivity interfering with number manipulation and mathematical problem solving. Several factor analytic models indicate that mathematics anxiety is rather a multidimensional than unique construct. However, the factor structure of mathematics anxiety has not been fully clarified by now. This issue shall be addressed in the current study. The Mathematics Anxiety Rating Scale (MARS) is a reliable measure of mathematics anxiety (Richardson and Suinn, 1972), for which several reduced forms have been developed. Most recently, a shortened version of the MARS (MARS30-brief) with comparable reliability was published. Different studies suggest that mathematics anxiety involves up to seven different factors. Here we examined the factor structure of the MARS30-brief by means of confirmatory factor analysis. The best model fit was obtained by a six-factor model, dismembering the known two general factors “Mathematical Test Anxiety” (MTA) and “Numerical Anxiety” (NA) in three factors each. However, a more parsimonious 5-factor model with two sub-factors for MTA and three for NA fitted the data comparably well. Factors were differentially susceptible to sex differences and differences between majors. Measurement invariance for sex was established.

## Introduction

High arousal and physiological reactivity in response to number manipulation are symptoms of mathematics anxiety (Richardson and Suinn, 1972; Dew et al., 1984; Faust, unpublished doctoral dissertation). They lead to avoidance of careers that require mathematical skills (Ashcraft and Faust, 1994; Ashcraft and Kirk, 2001; Hopko et al., 2001). Accordingly, women typically have higher values of mathematics anxiety than men (e.g., Devine et al., 2012) and mathematics anxiety differs across college majors (e.g., Preston, 1986, but see Hamza et al., 2011). However, mathematics anxiety may therefore contribute to impaired life functioning (e.g., Hopko et al., 2001).

Therefore quick and efficient identification of mathematics anxious persons by standardized instruments is important for intervention (see Richardson and Suinn, 1973 for an intervention study). For application in adults Richardson and Suinn (1972) constructed a measure of anxiety related to mathematics originally consisting of 98 items – the Mathematics Anxiety Rating Scale (MARS), which has been validated by several studies (Richardson and Suinn, 1972, 1973; Suinn et al., 1972; Brush, 1978; Morris et al., 1978). Since then, several studies developed abbreviated forms in order to reduce administration time or eliminate contaminated items that did not fit data (69 and 25 items by Alexander and Martray, 1989; 10 items by Ferguson, 1986; 34 items by Fujii, 1994; 12 items by Hopko, 2003; 67 items by Levitt and Hutton, 1984; 24 items by Plake and Parker, 1982; 94 items by Rounds and Hendel, 1980; see also **Table 1**). Summarizing results of Rounds and Hendel (1980), Alexander and Cobb (1987) and Alexander and Martray (1989), the authors of the original instrument themselves constructed a shortened scale consisting of 30 items, called the MARS30-brief (Suinn and Winston, 2003). They report a Cronbach’s alpha of 0.96 and test–retest reliabilty of 0.90 for this instrument and consider it to be comparable to the original 98 item scale.

**Table 1:** Factors of Mathematics Anxiety: overview of a variety of factor analytic studies on different versions of the MARS.

	Extraction method	Sample (%) females	MARS version	MTA		NA				
				LMA	EA	CA/OA	SRA	ENA	PA	PSA/AA
Rounds and Hendel, 1980	PCA Varimax rotation	350 (100)	94 items → 86 items	42 items		44 items				
Brush, 1978	PCA Varimax rotation	189 (51) students	94 items	45 items		31 items				
Suinn and Winston, 2003	PCA Oblique rotation	124 (51) psychology students	MARS30-brief	? items		? items				
Ferguson, 1986	PCA Varimax rotation	365 (?) college students	Phobos 30 items	? items	? items		10 items				
Alexander and Martray, 1989	PCA Varimax rotation	517 (?) college students	69 items → sMARS 25 items	15 items		5 items		5 items		
Resnick et al., 1982	PCA Varimax rotation	1045 ( ?) students	94 items → 30 items	19 Items			4 items	7 items	
Alexander and Cobb, 1987	PCA	197 (?) college students	MARS → 41 items	34 items		7 items				
Bessant, 1995	PCA ? Quartimax rotation	173 (59) psychology students	80 items canadian assimilation	52 items	? items	? items		? items	? items	? items
Jänen et al., 2006	PCA Orthogonal rotation CFA	666 (48) pupils	MARS-E (german)	5 items	5 items	4 items		6 items		
Plake and Parker, 1982	PCA Varimax rotation	170 (?) graduate students	MARS-R 24 items	16 items	8 items					
Hopko, 2003; Hopko et al., 2003	CFA	815 (51) students	MARS-R 24 items → AMAS12 items	8 Items	4 items					

*PCA, Principal component analysis; CFA, confirmatory factor analysis; MTA, Mathematics Test Anxiety; LMA, Learning Mathematics Anxiety; EA, Evaluation Anxiety; NA, Numerical Anxiety; SRA, Social Responsibility Anxiety; PA, Performance Anxiety; AA, Abstraction Anxiety; OA, Passive Observation Anxiety; CA, Course Anxiety; ENA, Everyday Numerical Anxiety; PSA, Problem Solving Anxiety.*

Several studies tried to disclose the factor structure of mathematics anxiety by applying factor analyses to different versions of the MARS. **Table 1** gives an overview of the factor structures obtained with different extraction methods, different samples, and test versions. Generally, a global 2-factor-structure is widely accepted (Rounds and Hendel, 1980; Alexander and Cobb, 1987). Different authors distinguish between two aspects of mathematics anxiety: “Mathematics Test Anxiety” (MTA) describing anxiety associated with learning for mathematics tests and being evaluated in mathematics, and “Numerical Anxiety” (NA) describing anxiety associated with the manipulation of numbers, basic arithmetic skills, and monetary decisions in everyday situations (see Rounds and Hendel, 1980; Alexander and Cobb, 1987).

Since mathematics test-related items evoke more anxiety than task- or course-related items (Alexander and Martray, 1989) some authors consider MTA to be the more important factor of mathematics anxiety and NA to play only a secondary role (Plake and Parker, 1982; Alexander and Martray, 1989). Therefore, Plake and Parker (1982) developed the MARS-R, which consists only of items concerning the MTA-factor. However, these authors still tried to base their measure on a multilevel model of mathematics anxiety and take into account that it is related to general state-, trait-, and test-anxiety. These authors described 2 subscales of the MARS-R or MTA: “Learning Mathematics Anxiety” (LMA), concerning learning for mathematics tests or homework, and “Mathematics Evaluation Anxiety” (EA), concerning mathematics tests and exams. This structure has been validated and replicated through confirmatory factor analysis by Hopko (2003). Interestingly, Alexander and Cobb (1987) assign a subset of items categorized as “Course Anxiety” to the MTA-factor, which are considered to be part of the NA scale by other authors (see **Table 1**). In summary, most studies report a one or two-factor structure of the MTA scale.

Regarding the factor structure of the global dimension NA, studies reveal a more fine-grained factor structure. The NA-factor is subdivided into “Everyday Numerical Anxiety” (ENA, Bessant, 1995), “Performance Anxiety” (PA, Bessant, 1995), “Social Responsibility Anxiety” (SRA, Resnick et al., 1982), “Observation Anxiety” (OA, Bessant, 1995), and “Problem Solving Anxiety” (PSA, Bessant, 1995), or “Abstraction Anxiety” (AA, Ferguson, 1986). ENA involves private calculations in everyday situations, while PA includes performance pressure induced by being told to solve mathematical problems. SRA concerns everyday life situations demanding social responsibility, e.g., memorizing figures for a driving license test. OA involves watching someone working on mathematical problems, with a calculator or on the blackboard. PSA/AA concerns abstract mathematical problem solving like equations or ratios. As can be depicted from **Table 1** a great variety of factor solutions has been obtained for the items pertaining to the NA scale.

In summary, the factor structure of mathematics anxiety remains unclear. Different reasons for this may be pointed out: In part this can be attributed to the large diversity of (i) extraction methods and (ii) item sets employed, and (iii) assignment of items to factors.

(i) First, a great variety of methods employed to investigate the covariance structure of the MARS can be observed (**Table 1**). While most authors have worked with exploratory methods for determining the number of factors necessary for accounting for a substantial proportion of variance (principal components analysis with different rotation methods, scree plot, fixation of the number of factors), only one study has so far used confirmatory factor analysis to investigate whether the MTA-factor consisted of one or two subfactors. On the one hand, exploratory methods imply dangers concerning overfactorization in the final item selection (Fabrigar et al., 1999). For instance, when the average item covariance is relatively low, the exploratory solution may reveal too many factors. On the other hand, relevant portions of the covariance structure of the original items set may be overseen when many items were eliminated, because they load on two or more separate factors simultaneously.

(ii) Secondly, the item selection in the different studies differed widely and was often not even explicitly reported. Some studies obtained their abbreviated versions not from the original 98-items scale, but from non-validated abbreviated item subsets. For instance, Rounds and Hendel (1980) ran a factor analysis over 94 out of the 98 original items, while Bessant (1995) used only 80 items and Alexander and Martray (1989) 69 items. Furthermore, Ferguson (1986) used 20 items that according to Rounds and Hendel (1980) loaded on one of the two factors MTA and NA, as well as 10 further items referring to abstract mathematical topics. The MARS-R of Plake and Parker (1982) consisted mainly of Items of the MTA-factor and, according to the authors, was designed for application in “*statistically related situations*” (Plake and Parker, 1982, p. 552). Problems with the lack of selection criteria may cumulate over studies when authors develop new reduced versions of the MARS from abbreviated item sets taken from the literature (Resnick et al., 1982; Hopko, 2003). As a result, the factor structure of abbreviated versions of the MARS may tap on very specific subset of the dimensions described in the literature (**Table 1**). To summarize, the widely varying item selections for different factor analyses may have led to very differing empirical and theoretical factor solution. In particular, some reduced version of the MARS may ignore important dimensions of mathematics anxiety and may be useful only for investigating specific aspects of this construct.

(iii)Third, the assignment of items to factors as described in the literature is very often incomplete. While, Plake and Parker (1982), Ferguson (1986), Alexander and Martray (1989), and Hopko (2003) reported exactly the assignment of all items surviving factor analysis to their respective factors as well as their loads in these factors, other authors have reported the assignment of items to factors only in an illustrative way. Therefore, it is possible that some items may have been assigned to different factors over different studies. Once more the unclear assignment of items to determined factors may lead to problems with the conceptual interpretation of the different dimensions of mathematics anxiety.

For these reasons further investigation on the factor structure of mathematics anxiety is still necessary. Specifically, it is relevant to determine (i) whether the traditional two-factor model by Rounds and Hendel (1980) is sufficient for describing the dimensionality of mathematics anxiety, (ii) whether these two factors as second-order factors can be dismembered into several smaller first-factors in a hierarchical CFA model and (iii) whether the second-order factors are necessary for describing the dimensionality of mathematics anxiety. In the present study we therefore examined and compared these three confirmatory factor analytic models. Especially, the MARS is probably still the most widely used mathematics anxiety questionnaire and the MARS30-brief is its present (abbreviated) version. While Richardson and Suinn (1972) report an internal consistency of 0.97 and a test–retest reliability of 0.85 for the MARS, Suinn and Winston (2003) report an internal consistency of 0.96 and test–retest reliability of 0.90 for the MARS-30 brief. According to the authors, validity data also support the comparability of the two measures. Thus, the MARS30-brief can be considered an economical equivalent of earlier versions of the MARS, which has been constructed under consideration of results from earlier studies, also accounting for their deficiencies in selection of samples and item sets. Therefore, disclosing its factor structure is of great empirical interest. To our knowledge, the factor structure of the current version of this diagnostic instrument has not been investigated with confirmatory factor analytic techniques yet. Therefore, in the present study the factor structure of the MARS30-brief was examined.

Establishing the factor structure of mathematics anxiety may help identifying, which aspects of the construct lead to the avoidance of careers requiring mathematical skills. When considering MTA and NA, it is of interest, whether the anxiety pertains to the performance of mathematics in itself, irrespective of the situation, or whether the anxiety is more strongly attributed to the test situation. The present study aims to evaluate, whether more sub-factors are necessary to gain an even closer picture of where and when the anxiety manifests for an individual. In particular it may be relevant, whether it already leads to the avoidance of learning math (LMA) or only to the avoidance of test situations (EA) or whether it leads to the avoidance of performing math in everyday life altogether (ENA) or only in situations of social responsibility (SRA).

Identifying, which aspects of math anxiety are most important for a person, is, however, of importance for successful intervention. Therefore, in the present study, after establishing the factor structure of the MARS30-brief, we will also assess individual differences in these sub-factors, particularly gender differences and differences between college majors. While gender differences and differences between college majors are commonly accepted for mathematics anxiety, only few studies have so far distinguished between different components of mathematics anxiety in these comparisons. This may in part be attributable to the fact that inconsistencies already arise, when taking only the two factors MTA and NA into account. According to Evans (2000) higher values in women were confirmed for both MTA and NA, whereas Baloǧlu and Koçak (2006) report higher MTA values in women, but higher NA values in men using a revised version of the MARS. Furthermore, it has been suggested based on different relationships of MTA and NA to age and attitudes toward mathematics in men and women that the factor structure of the mathematics anxiety may differ between men and women (Wilder, 2012). This has, however, not been confirmed using confirmatory factor analytic models. Therefore, we will establish measurement invariance prior to our gender comparisons, while the comparisons between majors need to remain exploratory due to small sample sizes in some groups. However, to the best of our knowledge, it has not been previously investigated, whether gender differences and differences across college majors, concern all sub-factors of MTA and NA or whether some factors are more sensitive for gender- and major-differences than others.

## Materials and Methods

### Participants

Participants were 491 students (330 women, 161 men, mean age: 21.78 years, SD = 4.05 years; range: 18–55 years) at the University of Salzburg. 162 of the participants (96 women, 66 men) were enrolled as psychology majors, 179 (124 women, 55 men) were enrolled as biology majors, 46 (26 women, 20 men) were enrolled as mathematics majors and 66 (55 women, 11 men) were enrolled as language majors. The remaining 38 participants were from other majors (e.g., education, history, geography) or did not provide any information about their major. The latter were not included in analyses comparing mathematics anxiety between majors.

### Ethics Statement

Participants were informed about the aims of the study and gave a written consent authorizing data processing for research purposes. Participation in the present study was voluntary. To assure anonymity in data processing, a numerical code was assigned to each participant. All methods conform to the Code of Ethics of the World Medical Association (Declaration of Helsinki).

The institutional guidelines of the University of Salzburg (Statutes of the University of Salzburg – see http://www.uni-salzburg.at/fileadmin/multimedia/Senat/documents/Satzung.pdf) state in §163 (1) that ethical approval is necessary for research on human subjects if it affects the physical or psychological integrity, the right for privacy or other important rights or interests of the subjects or their dependents. In §163, (2) it is stated that it is the responsibility of the PI to decide, whether (1) applies to a study or not. Therefore we did not seek ethical approval for this study. Since it was non-invasive and performed on healthy adult volunteers who gave their informed consent to participate, (1) did not apply.

### Measure

The MARS30-brief was developed by Suinn and Winston (2003) and is a 30-item instrument for individual or group-administration. Items represent mathematics-related situations that may cause anxiety in the respondent. The translation into German was conducted by the first author and corrected by her supervisors for administration in German-speaking participants (see **Table 2** for item examples). Participants reported their level of anxiety associated with a particular item by checking the corresponding token in a scale from “not at all” (0), “a little” (1), “a fair amount” (2), “much” (3) to “very much” (4). Therefore, scores in the individual items ranged from 0 to 4. The MARS30-brief was administered in an auditorium of the University of Salzburg to all participants at once. Measure instructions were read aloud by an experimenter; the same instructions were also printed on the first page of the MARS30-brief’s booklet. Instructing and administering the MARS30-brief took a total time of approximately 10 min. One and only one answer for each item was allowed. All participants conformed to these instructions – there were no missing data.

**Table 2:** Item examples for each factor.

EA1	Item1	Taking an examination (final) in a mathematics course^a^.
EA2	Item4	Thinking of an upcoming mathematics test on hour before.
LMA	Item10	Studying for a mathematics test.
ENA	Item20	Figuring out your monthly budget.
PA	Item21	Being given a set of numerical problems involving addition to solve on paper.
SRA	Item24	Being responsible for collecting dues for an organization and keeping track of the amount.

^a^*Copyright for the MARS test and all exemplary MARS items is owned by Richard M. Suinn, Ph.D., 808 Cheyenne Drive, Ft. Collins, CO 80525, USA. All rights reserved.*

### Analyses

**FIGURE 1:**
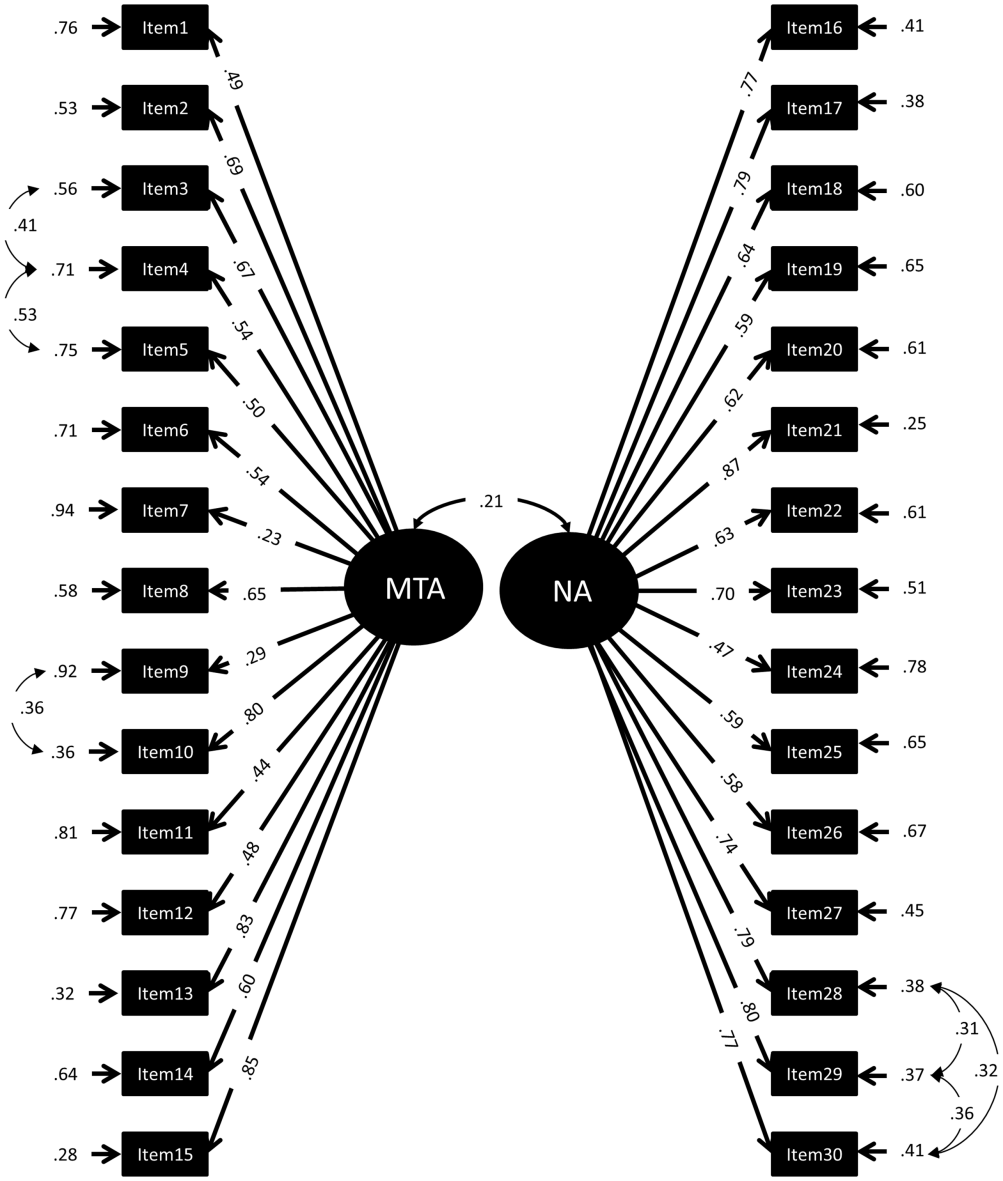
**Standardized Factor loadings, error variances, and correlations of a 2-factor-model of the MARS30-brief (Model 1).** MTA, Mathematical Test Anxiety (items 1–15); NA, Numerical Anxiety (items 16–30).

**FIGURE 2:**
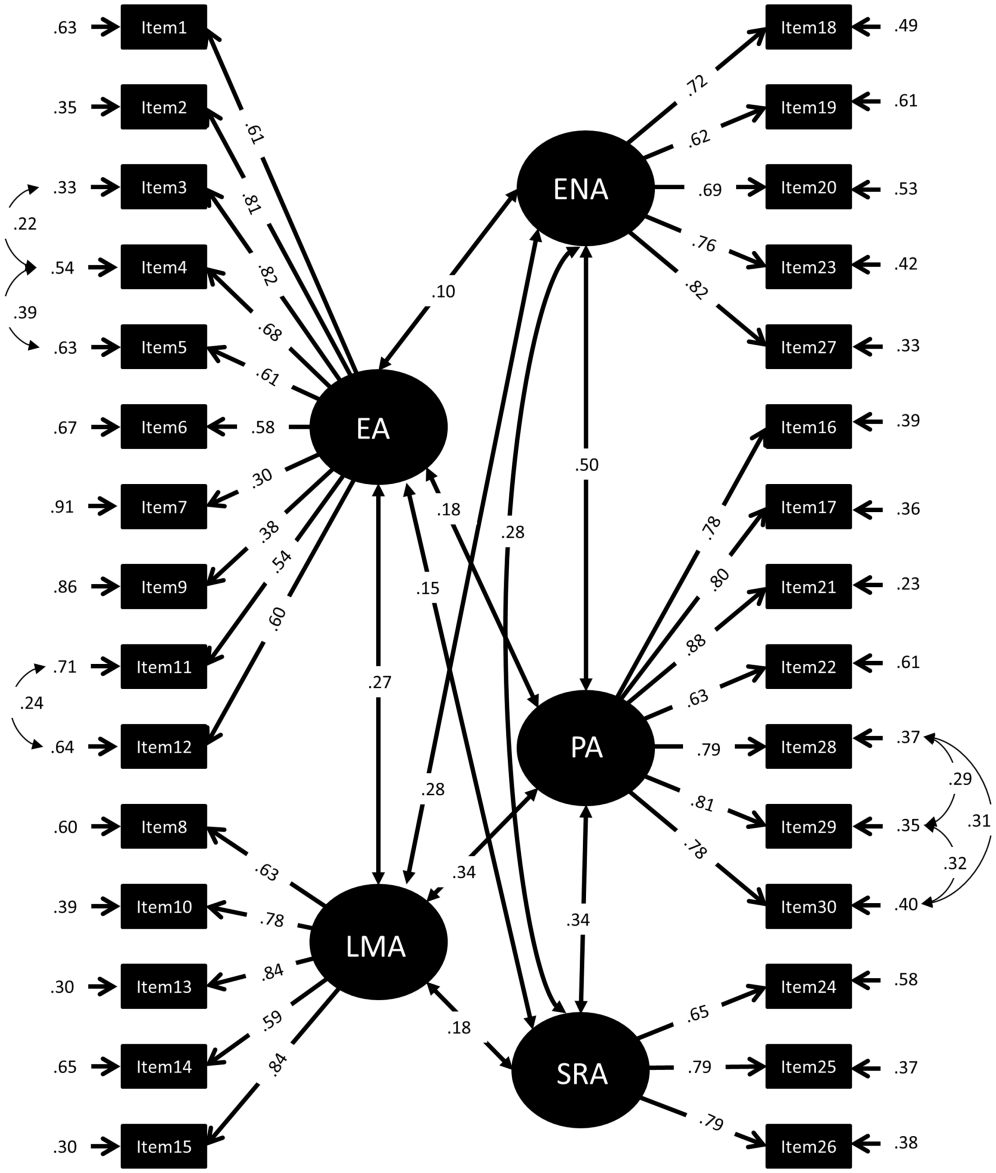
**Standardized Factor loadings, error variances, and correlations of a 5-factor-model of the MARS30-brief (Model 3).** EA, Evaluation Anxiety; LMA, Learning Mathematics Anxiety; ENA, Everyday Numerical Anxiety; PA, Performance Anxiety; SRA, Social Responsibility Anxiety.

**FIGURE 3:**
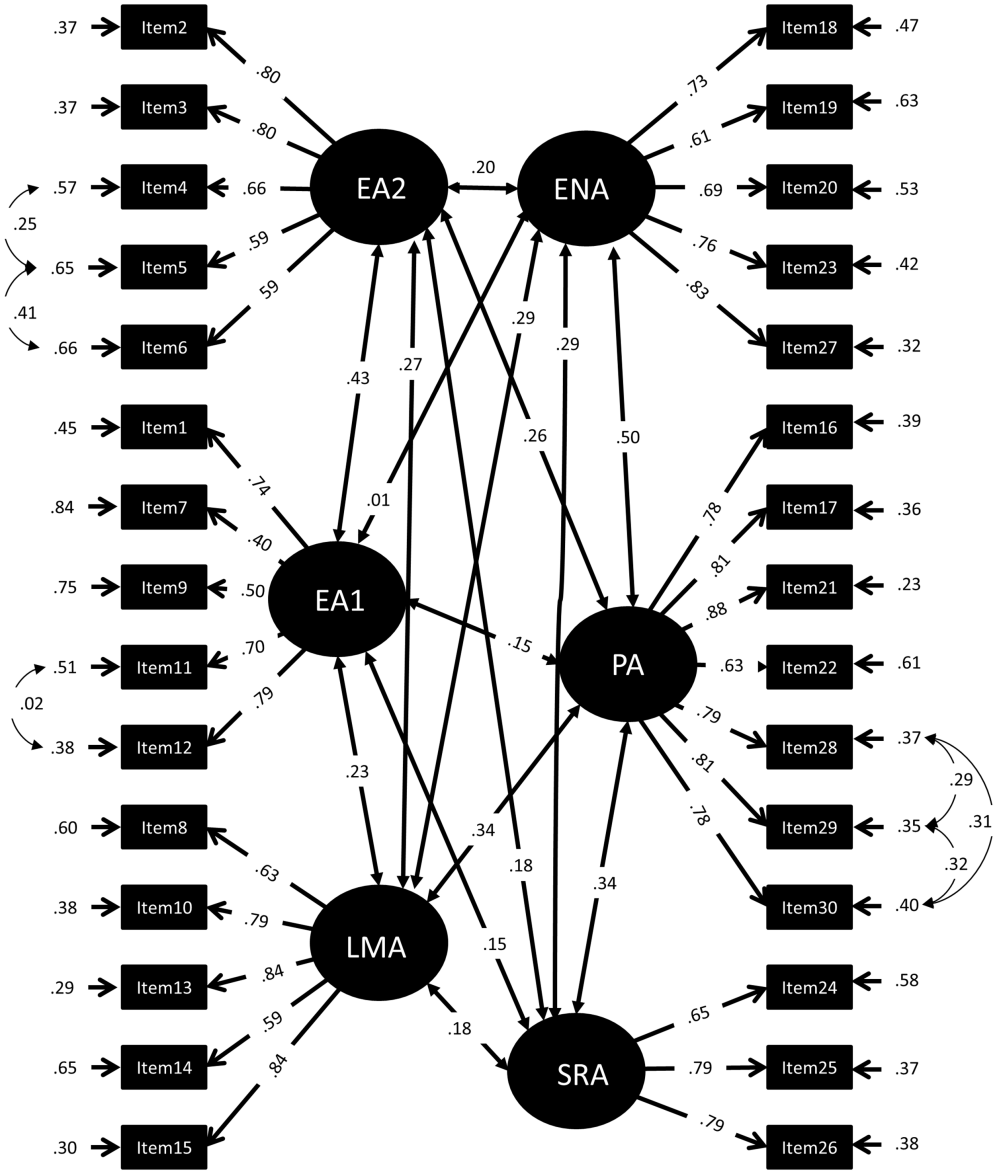
**Standardized Factor loadings, error variances, and correlations of a 6-factor-model of the MARS30-brief (Model 4).** EA1, Evaluation Anxiety proper (taking mathematics tests); EA2, Evaluation Anxiety (thinking about mathematics tests); LMA, Learning Mathematics Anxiety; ENA, Everyday Numerical Anxiety; PA, Performance Anxiety; SRA, Social Responsibility Anxiety.

To determine the factor structure of the MARS30-brief, a series of confirmatory factor models was calculated. We started the confirmatory factor analysis by examining the fit obtained for a default model (Model 0) for comparison, including only one global factor for mathematics anxiety (MARS). The first test model (Model 1) included two global factors, named “Mathematical Test Anxiety” (MTA) and “Numerical Anxiety” (NA). The assignment of items to factors MTA and NA was based on that reported by Rounds and Hendel (1980): We assigned items 1-15, all mentioning a mathematics test or exam to MTA, and items 16-30, all mentioning performing mathematics in everyday life to NA (see **Figure 1**). In a second model (Model 2), the factors MTA and NA were defined as second order factors (Marsh and Hocevar, 1985). Moreover, the first order factors EA and LMA were assigned to the second order factor MTA while the first order factors ENA, SRA, and PA loaded on the second order factor NA (see **Figure 2**). The assignment of items to EA, LMA, ENA, SRA, and PA was done as described in the literature (see Introduction and **Table 1**). All items referring to taking a mathematics examination were assigned to EA, all items referring to learning for a mathematics examination to LMA. All items referring to performing mathematics in everyday life (calculating a budget, reading a receipt) were assigned to ENA, all items referring to performing mathematics in a socially responsible role were assigned to SRA and all items simply referring to performing mathematics without giving a context (adding or dividing numbers on a paper) were assigned to PA. Item examples for each factor are listed in **Table 2**. The full list of items can be found in Suinn and Winston (2003). Note that Item 27 mentioned watching others work with a calculator, which would normally be assigned to OA. However, since this was the only item of this kind, it was assigned to ENA. The third model preserved only the first order factors of Model 2 but removed the second order factors (Model 3, see **Figure 3**).

**FIGURE 4:**
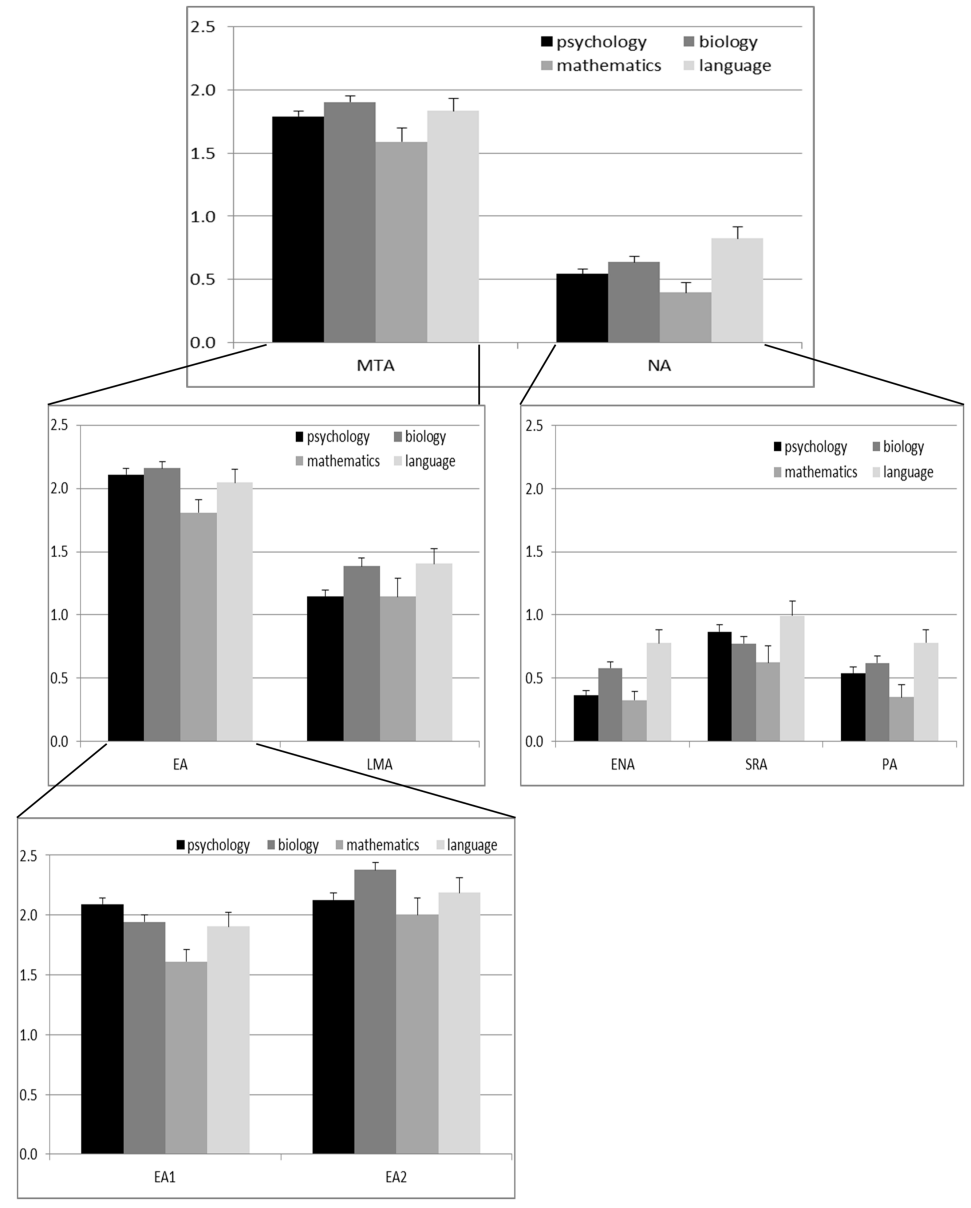
**Means ± SE for the different factors of mathematics anxiety split by major.** MTA, Mathematical Test Anxiety (items 1–15); NA, Numerical Anxiety (items 16–30). EA, Evaluation Anxiety; LMA, Learning Mathematics Anxiety; ENA, Everyday Numerical Anxiety; PA, Performance Anxiety; SRA, Social Responsibility Anxiety; EA1, Evaluation Anxiety proper (taking mathematics tests); EA2, Evaluation Anxiety (thinking about mathematics tests).

These 3 Models were constructed following strictly the description of Factors in the literature (compare **Table 1**). However, we realized that Items 2–6, albeit mentioning a mathematics exam or test, did not refer to actually taking that exam, but to thinking about the exam. In order to test, whether thinking about an examination represented a different component of MTA than actually taking an examination, a fourth model was tested including six instead of five first order factors (Model 4, compare **Figure 4**). Model 4 included the same factors as Model 3, with the exception that EA was split into EA1, being EA proper (taking an examination) and EA2 (thinking about an examination).

The same correlations between error terms were allowed in each model for items 3–5 (*thinking about a mathematics examination a day/hour/minutes before it takes place*) and items 28–30 (*being supposed to perform divisions/additions/multiplications*), because their wording was very similar, in fact differed only in one word.

Since we observed gender differences on some factor scores, but not others, we additionally tested the comparability of each model between men and women. First, model fit was obtained for each group. Then measurement invariance was established. Since total sample size is larger than 300, strict criteria were used for measurement invariance analysis as recommended in Chen (2007). Measurement invariance for loadings and residuals was assumed, if the reduction in CFI did not exceed 0.01 and the reduction in RMSEA did not exceed 0.015.

Model estimation and comparison as well as tests for multivariate normality were carried out using the lavaan package for R. To evaluate Model fit we chose the Comparative Fit Index (CFI), since we want to compare the fit between different models, the Tucker–Luis index TLI as a relative fit index, which is not affected by sample size and does penalize adding additional parameters to the model and the Root Mean Square Error of approximation (RMSEA) as a badness of fit index that takes model complexity into account. Models were accepted, if CFI was >0.95. Further statistical analyses were carried out using the software SPSS version 20. In particular, sub-factor scores were compared to each other using Wilcoxon and Friedman-tests. The total MARS score and the sub-factor scores were compared non-parametrically between genders using Mann–Whitney *U* tests and between majors using Kruskal–Wallis and Mann–Whitney *U* tests. For Mann–Whitney *U* tests between majors, the significance level was Bonferonni-corrected to 0.008.

## Results

### Normative Data

Participants reached an average total score of 36.83 (SD = 15.69, range: 1– 90). Ordinal alpha (based on the polychoric correlations) was 0.93. While this was lower than in the initial study of Suinn and Winston (2003) (α = 0.96), it can be considered satisfactory. A significant Kolmogorov–Smirnov test suggested that the MARS30-brief total score deviated from a normal distribution in the present study (*Z* = 1.46, *p* = 0.03). Average responses and standard deviations as well as ordinal alpha with this item deleted are presented for each item in **Table 3**. As can be depicted from **Table 3**, deletion of items does not change the reliability of the scale.

**Table 3:** Item statistics.

	*Mean*	*SD*	α if deleted	Skew	Kurtosis
Item 1	2.17	1.09	0.93	–0.16	–0.59
Item 2	1.74	1.08	0.93	0.17	–0.54
Item 3	2.58	1.14	0.93	–0.47	–0.65
Item 4	2.73	1.12	0.93	–0.60	–0.42
Item 5	2.75	1.17	0.93	–0.60	–0.54
Item 6	1.42	1.13	0.93	0.49	–0.65
Item 7	1.55	1.18	0.93	0.27	–0.83
Item 8	1.65	1.23	0.93	0.27	–0.90
Item 9	2.45	1.16	0.93	–0.48	–0.51
Item 10	1.35	0.96	0.93	0.54	–0.38
Item 11	1.91	0.96	0.93	–0.11	–0.86
Item 12	1.90	1.05	0.93	–0.16	–0.21
Item 13	0.70	1.04	0.93	1.36	1.20
Item 14	1.53	0.98	0.93	0.30	–0.50
Item 15	1.07	0.89	0.93	0.80	0.05
Item 16	0.53	0.98	0.93	1.95	3.09
Item 17	0.37	0.89	0.93	2.60	6.07
Item 18	0.44	0.99	0.93	2.37	4.77
Item 19	0.42	0.77	0.93	2.05	4.14
Item 20	0.75	1.07	0.93	1.44	1.28
Item 21	0.47	0.83	0.93	1.88	3.10
Item 22	0.98	1.07	0.93	0.96	0.14
Item 23	0.73	0.94	0.93	1.26	1.13
Item 24	1.02	1.01	0.93	0.83	0.09
Item 25	0.76	0.93	0.93	1.14	0.72
Item 26	0.75	0.90	0.93	1.10	0.67
Item 27	0.23	0.64	0.93	3.27	11.54
Item 28	0.75	0.96	0.93	1.21	0.81
Item 29	0.53	0.84	0.93	1.71	2.73
Item 30	0.58	0.90	0.93	1.60	2.08

### Confirmatory Factor Analytic Models

The covariance structure presented by the 30 items of the MARS30-brief did not follow a multivariate normal distribution based on Mardias test for multivariate normality (*X^2^* = 384.55, *p* < 0.001) since neither multivariate skewness (β_*1*_ = 207.87; *X^2^* = 17010.56, *p* < 0.001) nor multivariate kurtosis (β_*2*_ = 1347.63; *Z* = 98.01, *p* < 0.001) were within an acceptable range. As indicated by significant Kolmogorov–Smirnov tests, each items deviated from a univariate normal distribution as well (*p* < 0.001). For this reason the CFA-model including all 30 items have been estimated with the unweighted least squares method of estimation (Bentler and Dudgeon, 1996; Schumacker and Lomax, 2004). Since ordinal data were obtained on a Likert scale, CFA-models were based on the polychoric correlation matrix and asymptotic covariance matrix.

In a first step, we evaluated the fit of the default model, with all items assigned to one factor MARS (Model 0). This model did not obtain a satisfactory model fit (compare **Table 4**), indicating that mathematics anxiety as assessed with the MARS30-brief is comprised of more than one factor.

**Table 4:** *X*^2^ and fit indices for the reported models.

	Model fit					Model comparison				
	*Df*	*X^2^*	CFI	TLI	RMSEA	Model	Δ*X*^2^	Δdf	*P*	
Model 0 (1 Factor)	399	4034.19	0.87	0.86	0.14					Reject
Model 1 (2 Factors)	398	2471.85	0.93	0.92	0.10	To Model 0	1562.30	1	<0.001	Reject
Model 2 (2-stages)	393	1702.98	0.95	0.95	0.08	Discarded due to negative error variances				
Model 3 (5 Factors)	389	1617.09	0.96	0.95	0.08	To Model 1	854.75	4	<0.001	Accept
Model 4 (6 Factors)	384	1373.70	0.97	0.96	0.07	To Model 3	243.39	5	<0.001	Accept

*Model 1: two-factor model adapted from Rounds and Hendel (1980); Model 2: two stage model with two second order and six first order factors; Model 3: six-factor model with no second order factors. CFI, Comparative Fit Index; TLI, Tucker-Lewis Index; RMSEA, Root Mean Square Error of Approximation, for all models p < 0.05.*

In a second step different factor structures were tested and compared to the default model. To examine the two-factor structure reported by Rounds and Hendel (1980), we assigned items 1–15 all mentioning a mathematics test or exam to MTA and items 16–30 to NA (Model 1, **Figure 1**). The high *X*^2^ value and borderline fit indices associated with Model 1 point out that this two-factor model cannot account for the covariance structure of data satisfactorily (**Table 4**). This suggests that the structure of mathematics anxiety is more fine-grained than a simple distinction of MTA and NA constructs. Importantly, however, the sum of scores for MTA (Items 1–15; 27.50 ± 9.85) were significantly higher than the sum or scores for NA (items 16–30; 9.33 ± 8.81; *Z* = 18.75; *p* < 0.001). Ordinal alphas of MTA and NA were both 0.89.

In Model 2 the two-factor structure was dismembered into a hierarchical CFA structure with the two original MTA and NA factors as second order factors. To second order factor MTA the first order factors EA and LMA were assigned and to the second order factor NA the first order factors ENA, PA, and SRA. This model resulted in negative error variances, suggesting a bad fit for the data and was therefore discarded. Therefore, Model 3 included only the five factors EA, LMA, ENA, PA, and SRA, but the second order factors MTA and NA were removed (**Figure 2**). The *X^2^* value associated with Model 3 was significantly lower than that of Models 1 and model fit was much better. This suggests that a non-hierarchical five-factor model describes the factor structure of the MARS30-brief better than the two-factor solution in Model 1. Ordinal alphas of the 5 factors in the CFA-model were 0.86, 0.86, 0.84, 0.89 and 0.96 for EA, LMA, ENA, SRA, and PA, respectively. Average scores for EA (2.11 ± 0.71) were significantly higher than for LMA (1.26 ± 0.81; *Z* = 16.75, *p* < 0.001). Furthermore, the scores on the sub-factors of NA did differ significantly from each other as indicated by a Friedman test (*X*^2^ = 99.57, *p* < 0.001). As indicated by Wilcoxon comparisons (all *Z* > 4.16, all *p* < 0.001), ENA (0.52 ± 0.03) was significantly lower than PA (0.60 ± 0.04) and SRA (0.84 ± 0.04), while SRA was significantly higher than PA and ENA.

Furthermore we tested, whether model fit could be further improved, by dismembering the EA factor into EA1 (taking an examination) and EA2 (thinking about an examination), which has not been described in the literature before (**Figure 3**). Indeed, the model fit obtained by this model (Model 4) was best and the *X*^2^ value was significantly lower than in Model 3. This suggests that other than described in the literature the MTA factor was comprised of more than two components, since taking an examination and thinking about a examination comprised different sub-factors of MTA. Ordinal alphas of EA1 and EA2 were 0.73 and 0.83, respectively. Average scores for EA1 (1.99 ± 0.78) were significantly higher than average scores for EA2 (2.25 ± 0.85; *Z* = 5.85, *p* < 0.001).

Model comparisons are also displayed in **Table 4** indicating that Model fit was significantly improved in each step. **Tables 5** and **6** provide the factor pattern, coefficients and factor correlations for Models 3 and 4.

**Table 5:** Factor pattern and structure coefficients for Models 3 (5 Factor) and 4 (6 Factor).

	Model 3 (5 Factor)		Model 4 (6 Factor)	
Item	Factor	Coefficient	Factor	Coefficient
Item 2	EA	0.35	EA1	0.37
Item 3	EA	0.32	EA1	0.37
Item 4	EA	0.54	EA1	0.57
Item 5	EA	0.63	EA1	0.65
Item 6	EA	0.67	EA1	0.66
Item 1	EA	0.63	EA	0.45
Item 7	EA	0.91	EA	0.84
Item 9	EA	0.86	EA	0.75
Item 11	EA	0.71	EA	0.51
Item 12	EA	0.63	EA	0.38
Item 8	LMA	0.60	LMA	0.60
Item 10	LMA	0.39	LMA	0.38
Item 13	LMA	0.30	LMA	0.29
Item 14	LMA	0.65	LMA	0.65
Item 15	LMA	0.30	LMA	0.30
Item 18	ENA	0.49	ENA	0.47
Item 19	ENA	0.61	ENA	0.63
Item 20	ENA	0.53	ENA	0.53
Item 23	ENA	0.42	ENA	0.42
Item 27	ENA	0.33	ENA	0.32
Item 16	PA	0.39	PA	0.39
Item 17	PA	0.36	PA	0.35
Item 21	PA	0.23	PA	0.23
Item 22	PA	0.61	PA	0.61
Item 28	PA	0.37	PA	0.37
Item 29	PA	0.35	PA	0.35
Item 30	PA	0.40	PA	0.40
Item 24	SRA	0.58	SRA	0.58
Item 25	SRA	0.37	SRA	0.37
Item 26	SRA	0.38	SRA	0.37

*EA, Evaluation Anxiety; EA1, Evaluation Anxiety proper (taking an examination); EA2, thinking about an examination; LMA, Learning Math Anxiety; ENA, Everyday Numerical Anxiety; PA, Performance Anxiety; SRA, Social Responsibility Anxiety.*

**Table 6:** Latent Factor correlations for Model 3 (5 Factors, below diagonal) and Model 4 (6 Factors, above diagonal).

		EA		LMA	ENA	PA	SRA
		EA1	EA2				
**EA**	**EA1**		0.43	0.23	0.01	0.15	0.15
	**EA2**			0.27	0.20	0.26	0.18
**LMA**		0.27			0.29	0.34	0.18
**ENA**		0.10		0.28		0.50	0.29
**PA**		0.18		0.34	0.50		0.34
**SRA**		0.15		0.18	0.28	0.34	

*EA, Evaluation Anxiety; EA1, Evaluation Anxiety proper (taking an examination); EA2, thinking about an examination; LMA, Learning Math Anxiety; ENA, Everyday Numerical Anxiety; PA,Performance Anxiety; SRA, Social Responsibility Anxiety.*

### Gender Differences

An analysis of measurement invariance was conducted on Models 3 and 4 to see whether the same factor structure can be obtained for men and women. First, Models 3 and 4 provided comparably good fit for both the male (Model 3: *X*^2^ = 726.10, df = 389, CFI = 0.96, TLI = 0.96, RMSEA = 0.07; Model 4: *X*^2^ = 676.65, df = 384, CFI = 0.97, TLI = 0.96, RMSEA = 0.07) and female subsample (Model 3: *X*^2^ = 1316.53, df = 389, CFI = 0.95, TLI = 0.95, RMSEA = 0.09; Model 4: *X*^2^ = 1110.81, df = 384, CFI = 0.96, TLI = 0.96, RMSEA = 0.07). Results for different types of measurement invariance are displayed in **Tables 7** and **8**. While each additional constraint significantly reduced the *X*^2^ value of the model, model fit remained acceptable until the last step. Thus, mean factor scores can be compared between men and women.

**Table 7:** Measurement invariance between men and women for Model 3 (5 Factor).

	Model fit				Model comparison						
	Df	*X^2^*	CFI	RMSEA	Model	Δ*X*^2^	Δdf	ΔCFI	ΔRMSEA	*P*	
Configural	778	2042.60	0.96	0.08							Accept
Loadings	803	2197.60	0.95	0.08	To configural	154.94	25	<0.01	<0.015	<0.001	Accept
Intercepts/residuals	888	2401.60	0.95	0.08	To loadings	204.01	85	<0.01	<0.015	<0.001	Accept
Means	893	2894.30	0.93	0.10	To residuals	492.74	6	>0.01	> 0.015	<0.001	Reject

**Table 8:** Measurement invariance between men and women for Model 4 (6 Factor).

	Model fit				Model comparison						
	Df	*X^2^*	CFI	RMSEA	Model	Δ *X*2	Δ df	Δ CFI	Δ RMSEA	*P*	
Configural	768	1787.50	0.97	0.07							Accept
Loadings	792	1904.60	0.96	0.08	To configural	117.16	24	<0.01	<0.015	<0.001	Accept
Intercepts/residuals	876	2133.40	0.96	0.08	To loadings	228.74	84	<0.01	<0.015	<0.001	Accept
Means	882	2632.00	0.94	0.09	To residuals	498.67	6	> 0.01	= 0.013	<0.001	Reject

As described in the literature, the MARS total score was significantly higher in women (38.48 ± 15.67) than in men (33.42 ± 15.22) (*Z* = 3.18, *p* = 0.001). Gender differences were only observed in the first 15 items (MTA; *Z* = 4.40, *p* < 0.001), but not in the second 15 items (NA; *Z* = 0.33, *p* = 0.74). Gender differences were furthermore confirmed for all sub-factors of MTA (LMA, EA, EA1, and EA2; all *Z* > 2.48, all *p* < 0.05), but only for the sub-factor PA of NA (*Z* = 1.97, *p* < 0.05), not for ENA and SRA (both *Z* < 0.79, both *p* > 0.43).

### Differences Between Majors

Due to small sample sizes in some subgroups analyses of measurement invariance across majors could not be conducted. Therefore the following results are exploratory.

The MARS total score differed significantly between major subjects (*X*^2^ = 15.70, *p* = 0.001). Mathematics majors had significantly lower values than biology and language majors (all *Z* > 3.15, all *p* < 0.002). Psychology majors had by trend higher values than mathematics majors (*Z* = 2.32, *p* = 0.02), but by trend lower values than biology or language majors (both *Z* > 1.96, both *p* < 0.05). Biology and German majors had comparable values (*Z* = 0.55, *p* = 0.58). Major subject had a significant impact on both MTA and NA. However, Mann–Whitney *U* tests indicated that while for MTA highest scores were obtained by biology majors (significantly higher than mathematics majors, *Z* = 2.74, *p* = 0.002), for NA highest scores were obtained by language majors (significantly higher than psychology and mathematics majors, *Z* = 3.79, *p* < 0.001; compare **Figure 4**). Significant differences between the majors were also observed for all sub-factors of MTA and NA (all *X*^2^ > 8.64, all *p* < 0.05). Interestingly, for both EA and LMA the highest scores were obtained by biology majors. However, when split between EA1 and EA2, the highest scores for EA1 were obtained by psychology majors (significantly higher than mathematics majors, *Z* = 4.07, *p* < 0.001; not different from biology majors, *Z* = 1.57, *p* = 0.12), whereas only for EA2 the highest scores were obtained by biology majors (significantly higher than psychology majors, *Z* = 3.06, *p* = 0.002). For ENA, PA, and SRA, however, the highest scores were obtained by language majors.

## Discussion

As can be depicted from **Table 1** a great variety of factor solutions of mathematics anxiety exists. A global two-factor structure consisting of MTA and NA is widely accepted (Rounds and Hendel, 1980; Alexander and Cobb, 1987). However several studies report different sets of smaller factors. In the present study four factor analytic models were carried out in order to disclose the factor structure of mathematics anxiety, in particular the MARS30-brief. We wanted to determine (i) whether the traditional two-factor structure (i.e., MTA and NA as first order factors), first described by Rounds and Hendel (1980) is sufficient for describing the dimensionality of mathematics anxiety, (ii) whether MTA and NA can be dismembered into the first-order factors EA, LMA, ENA, PA, and SRA in a hierarchical CFA model and (iii) whether MTA and NA are necessary for describing the dimensionality of mathematics anxiety and (iv) whether EA could be further subdivided into EA1 (taking mathematics examinations) and EA2 (thinking about mathematics examinations). Furthermore, the present study aimed to evaluate, whether gender differences and differences across majors were comparable across all factors of mathematics anxiety and whether as a consequence the factor structure was comparable between men and women.

Our confirmatory factor models showed that (i) the two-factor structure was only borderline acceptable as description of the MARS30-brief in a single model, (ii) a hierarchical CFA factor structure having MTA and NA as second order factors described data equally well as the non-hierarchical five-factor model including EA, LMA, ENA, PA, and SRA. However, the best fit was obtained for a model including the six first order factors EA1, EA2, LMA, ENA, PA, and SRA. In the following these results will be discussed in more detail. Contrary to previous studies (Evans, 2000; Baloǧlu and Koçak, 2006), gender differences with higher scores in women were observed only for MTA, not for NA, however, equally for all sub-factors of MTA (EA, EA1, EA2, and LMA). These differences were, however, not attributable to differences in the factor structure of the MARS between men and women, since measurement invariance for gender could be established. Differences across majors were observed for MTA, NA as well as all sub-factors except SRA. However, while the highest scores for MTA were obtained by biology majors, the highest score for NA were obtained by language majors. Furthermore, within the MTA, but not the NA, sub-factors differences were observed, with psychology majors showing the highest scores for EA1, while biology majors showed the highest scores for EA2 and LMA.

In Model 1 we examined the 2-factor structure consisting of MTA and NA which was reported by Rounds and Hendel (1980) for the original MARS and assumed by Suinn and Winston (2003) for the MARS30-brief. This assumption about the factor structure of the MARS30-brief was also supported by our descriptive and normative item characteristics. MTA and NA differ not only in their mean item scores, which are lower for factors of NA (compare also Alexander and Martray, 1989), but also in their distribution characteristics. While the 15 items of MTA do not deviate from a multivariate normal distribution, thereby replicating findings of Hopko (2003), items of NA violate the assumption of multivariate normality. Although these results may in part have been caused by the order of items, the strength of effects suggests that another reason is more plausible. One possible explanation is the increased relevance of test situations in comparison with everyday arithmetical problems in the population tested in this study, i.e., students. This does not mean that the average scores in NA may be necessarily low and present a non-normal distribution in every population. It could be suggested that the average scores in this part of the MARS30-brief scale should be higher in populations for whom the relevance of calculation in daily living situations is higher such as by bank workers or tradesmen. This assumption was in part confirmed by our data. On the one hand, gender differences were only apparent for MTA, but not NA. On the other hand different majors showed highest values for MTA (biology) and NA (language), indicating a higher relevance of mathematical tests for science majors, but higher relevance of everyday mathematical calculations for non-science majors. The fact that biology students show such a high degree of MTA also suggests that not all science majors can be grouped together in their evaluation of mathematics anxiety. This has, however, been done in previous comparisons of mathematics anxiety between college majors (Hamza et al., 2011). Such a grouping of all science majors may cause an over- or under-estimation of mathematics anxiety differences between majors and may cost some majors (e.g., biology majors) the necessary attention they require in dealing with their mathematics anxiety.

However, using confirmatory factor analytic techniques, we could not confirm the results obtained previously with principal components analysis and a fixed number of factors. Although taking into account error covariances, the *X*^2^ value and fit indices of Model 1 were not satisfactory. Thus, our data clearly suggest that the two global factors MTA and NA are not sufficient for describing the factor structure of the MARS30-brief, but that its factor structure has more facets.

As an alternative hypothesis (ii) one could assume that the two dimensions MTA and NA perform better describing the covariance between the more specific first order factors. Therefore, in Model 2 the MTA – NA structure was dismembered into several smaller factors in a two stage two-factor model. This model, however, had to be discarded due to negative error variances, providing support for Models 3 and 4.

Model 3 and 4 differentiate better between different aspects of mathematics anxiety. Our factors EA and LMA replicate Hopko (2003), ENA, and PA have already been reported by Bessant (1995) and SRA by Resnick et al. (1982). Our Model 3 therefore includes all factors reported in the literature except OA and AA. However, OA was represented within the MARS30-brief by only 1 item (Item27), while the items of the construct AA were not originally contained in the MARS. It is to note that in contrast with findings of Bessant (1995), but replicating findings of Resnick et al. (1982), a strong association between factors ENA and PA was observed. Since Bessant (1995) forced factors ENA and PA as well as item residuals to be uncorrelated, it remains an open question, whether this association is generally high or only in our specific population.

However, contrary to the literature, the best model fit was obtained when further splitting EA into two factors capturing different aspects of EA, i.e., EA proper (taking and examination) as opposed to EA2 (just thinking about an examination). These two factors particularly seemed to induce different levels of anxiety across different majors with psychology majors showing particularly high values on EA1, but low values on all other aspects of mathematics anxiety. Furthermore, the correlation between EA1 and ENA was almost 0, whereas the correlation between EA2 and ENA was of moderate strength, suggesting different qualities of these two factors. We do note, however, that the results on differences between college majors need to be interpreted with care, since measurement invariance could not be established for these groups due to small sample sizes. Since Model fit of Model 3 is also acceptable and Model 3 is more parsimonious, Model 3 is probably the most practical model for research questions not evaluating differences between college majors.

In summary, the present findings on mathematics anxiety do not support the view that it can be reduced to MTA as has been suggested by Plake and Parker (1982) and Hopko (2003). Dew and Galassi (1983) and Dew et al. (1984) found that mathematics anxiety measures are more highly related to each other than to measures of test anxiety and therefore still reflect different aspects of personality. For a successful career it could rather be important to reduce SRA and PA to a reasonable and productive value. Through such an approach of differential diagnosis, intervention can target especially those constructs with high scores.

## Conflict of Interest Statement

The authors declare that the research was conducted in the absence of any commercial or financial relationships that could be construed as a potential conflict of interest. The reviewer CP and handling Editor declared a current collaboration and the handling Editor states that the process nevertheless met the standards of a fair and objective review.
